# Predicted savings to the UK National Health Service from switching to generic antiretrovirals, 2014–2018

**DOI:** 10.7448/IAS.17.4.19497

**Published:** 2014-11-02

**Authors:** Andrew Hill, Teresa Hill, Sophie Jose, Anton Pozniak

**Affiliations:** 1 Department of Pharmacology and Therapeutics, University of Liverpool, Liverpool, UK; 2 Department of Infection and Population Health, University College, London, UK; 3 St Stephens Centre, Chelsea and Westminster Hospital, London, UK

## Abstract

**Introduction:**

In other disease areas, generic drugs are normally used after patent expiry. Patents on zidovudine, lamivudine, nevirapine and efavirenz have already expired. Patents will expire for abacavir in late 2014, lopinavir/r in 2016, and tenofovir, darunavir and atazanavir in 2017. However, patents on single-tablet regimens do not expire until after 2026.

**Methods:**

The number of people taking each antiretroviral in the UK was estimated from 23,655 individuals in the UK CHIC cohort (2012 database). Costs of patented drugs were taken from the British National Formulary database, assuming a 30% discount. Costs of generic antiretrovirals were estimated using an 80% discount from patented prices, or actual costs where available. Two options were analysed: 1 – all patients use single-tablet regimens and patented versions of drugs; prices remain stable over time; 2 – all people switch from patented to generic drugs when available, after patent expiry (dates shown above).

**Results:**

There were an estimated 67,000 people taking antiretrovirals in the UK in 2014, estimated to rise by 8% per year until 2018 (in line with previous rises). The most widely used antiretrovirals in the CHIC cohort were tenofovir (TDF) (75%), emtricitabine (FTC) (69%), efavirenz (EFV) (39%), lamivudine (3TC) (23%), abacavir (ABC) (18%), darunavir (DRV) (21%) and atazanavir (ATV) (16%). The predicted annual UK cost of generic ABC/3TC/EFV (three generic tablets once daily) was £1018 per person-year. Costs of patented single-tablet regimens ranged from £5000 to £7500 per person-year. Assuming continued use of patented antiretrovirals in the UK, the predicted total national costs of antiretroviral treatment were predicted to rise from £425 million in 2014 to £459 m in 2015, £495 m in 2016, £536 m in 2017 and £578 m in 2018. With a 100% switch to generics, total predicted costs were £337 m in 2014, £364 m in 2015, £382 m in 2016, £144 m in 2017 and £169 m in 2018. The total predicted saving over five years from a switch to generics was £1.1 billion.

**Conclusions:**

Systematic switching from patented to generic antiretrovirals could potentially save approximately £1.1 billion in the UK over the next five years, compared with continued use of patented versions: this money could be spent on urgently needed HIV prevention programmes. Similar savings are feasible for other European countries, given parallel patent expiry dates. More detailed economic evaluation is required to show when patented single-tablet regimens provide value for money, compared to bioequivalent generic versions of 3–4 pills once daily.

